# Phylogenomics of Tick Inward Rectifier Potassium Channels and Their Potential as Targets to Innovate Control Technologies

**DOI:** 10.3389/fcimb.2021.647020

**Published:** 2021-03-19

**Authors:** Perot Saelao, Paul V. Hickner, Kylie G. Bendele, Adalberto A. Pérez de León

**Affiliations:** ^1^ USDA-ARS Knipling-Bushland U.S. Livestock Insects Research Laboratory, Kerrville, TX, United States; ^2^ Veterinary Pest Genomics Center, Kerrville, TX, United States

**Keywords:** phylogenomic, tick, Kir, Acari, evolution

## Abstract

This study was conducted to enhance the identification of novel targets to develop acaricides that can be used to advance integrated tick-borne disease management. Drivers for the emergence and re-emergence of tick-borne diseases affecting humans, livestock, and other domestic animals in many parts of the world include the increased abundance and expanded geographic distribution of tick species that vector pathogens. The evolution of resistance to acaricides among some of the most important tick vector species highlights the vulnerability of relying on chemical treatments for tick control to mitigate the health burden of tick-borne diseases. The involvement of inward rectifier potassium (Kir) channels in homeostasis, diuresis, and salivary gland secretion in ticks and other pests identified them as attractive targets to develop novel acaricides. However, few studies exist on the molecular characteristics of Kir channels in ticks. This bioinformatic analysis described Kir channels in 20 species of hard and soft ticks. Summarizing relevant investigations on Kir channel function in invertebrate pests allowed the phylogenomic study of this class of ion channels in ticks. How this information can be adapted to innovate tick control technologies is discussed.

## Introduction

Ticks (Acari) are obligate blood feeding parasites and vectors of a diverse array of pathogens including bacteria, protozoa, and viruses that cause diseases among humans, livestock, and other domestic animals (Sonenshine and Roe, 2013). The health burden of tick-borne disease (TBD) increased globally (Paules et al., 2018; Madison-Antenucci et al., 2020). In the United States alone, the number of human TBD cases more than doubled increasing from 22,527 to 48,610 between 2004 and 2016 (Rosenberg et al., 2018). Several species of hard and soft ticks are vectors of the diverse pathogens causing tick-borne diseases (Brites-Neto et al., 2015). Recent studies have implicated ticks in Alpha-Gal Syndrome, a potentially life-threatening allergy to red meat that is induced by the sugar alpha-gal from a feeding tick (Crispell et al., 2019). The application of technological advances is helping explain the global diversity of ticks and their ability to transmit pathogens (Dantas-Torres, 2018; Yang and Han, 2018).

Drivers for the emergence and re-emergence of tick-borne diseases affecting humans, livestock, and other domestic animals in many parts of the world include the increased abundance and expanded geographic distribution of tick species that vector pathogens. *Dermacentor variabilis*, the principal vector of *Rickettsia rickettsii* causing Rocky Mountain spotted fever in North America, expanded its range northward into Canada surviving the milder winters associated with variability in climatic patterns (Sonenshine, 2018). Due to its expanding geographic distribution and vector biology, *Ixodes scapularis* has become one of the most important disease vectors in North America where it transmits *Borrelia burgdorferi*, *Babesia microti*, and Powassan virus, causing Lyme disease, human babesiosis, and Powassan virus disease, respectively (Eisen and Eisen, 2018).

In addition, *Amblyomma americanum* and *Amblyomma maculatum* have expanded their range northward, extending the geographic range of additional tick species that threaten livestock (Sonenshine, 2018). Detection of the Asian longhorned tick, *Haemaphysalis longicornis*, in the United States (U.S.) in 2017 highlights the involvement of invasive tick species in the emergence of tick-borne diseases (Rainey et al., 2018). *Ha. longicornis* is a known vector of pathogens that affect humans, livestock, and other domestic animals (Beard et al., 2018). Control of tick populations, treatment of host infestations, and reduction of exposure to tick bites are critical for tick-borne disease management (Pérez De León et al., 2014).

The evolution of resistance to acaricides among some of the most important tick vector species highlights the vulnerability of relying on chemical treatments for tick control to mitigate the health burden of tick-borne diseases. In the U.S. for example, bovine babesiosis, or cattle tick fever, was eradicated by eliminating the one-host fever tick vectors, *Rhipicephalus microplus* and *R*. *annulatus*, through efforts of the Cattle Fever Tick Eradication Program including the systematic treatment of cattle with acaricides (Guerrero et al., 2014). However, the spread of resistance to acaricides approved for use by the Cattle Fever Tick Eradication Program among fever ticks causing outbreaks raises the specter for the emergence of bovine babesiosis in the U.S. (Thomas et al., 2020). Resistance to commonly used commercial acaricides in the three-host ticks *R*. *sanguineus* and *Amblyomma mixtum*, which are known vectors of human tick-borne diseases, stresses the need to innovate tick control technologies (Rodriguez-Vivas et al., 2017; Higa et al., 2020).

Discovering unique molecular targets facilitates the development of safer acaricides with new modes of action (Meng and Sluder, 2018). Previous research identified the inward rectifier potassium (Kir) channels as attractive targets to develop novel acaricides because of their involvement in homeostasis, diuresis, and salivary gland secretion in ticks and other pests (Li et al., 2019; Li et al., 2020). However, few studies exist on the molecular characteristics of Kir channels in ticks. Here, a bioinformatic analysis described Kir channels in 20 species of hard and soft ticks. Summarizing relevant investigations on Kir channel function in invertebrate pests allowed the phylogenomic study of this class of ion channels in ticks. The adaptation of this information to innovate tick control technologies is discussed.

## Kir Channel Structure and Genome Repertoires

Inward rectifier potassium (Kir) channels were named due to their ability to facilitate the inward movement of K+ ions at hyperpolarizing membrane voltages more readily than the outward movement of K+ ions at depolarizing membrane voltages, a function which has been compared to an electrical diode wherein current flows in only one direction (Nishida and Mackinnon, 2002; Tao et al., 2009). The S4 voltage sensor region found in voltage-gated K^+^, Na^+^, and Ca^2+^ channels is absent in Kir channels; therefore, Kir channels are refractory to changes in membrane potential associated with the inward movement of other cations (Nishida and Mackinnon, 2002; Hibino et al., 2010). The outward movement of K^+^ is inhibited by intracellular cations and polyamines that enter the pore from the cytoplasmic side but cannot pass through, thus blocking the channel and preventing the outward flow of K^+^ (Nishida and Mackinnon, 2002; Hibino et al., 2010). These properties allow Kir channels to maintain resting membrane potential and regulate action potential duration (Hibino et al., 2010).

Kir channels are formed by the assembly of four identical or similar protein subunits, each with two transmembrane domains flanking a re-entrant loop region forming the channel pore, and N- and C-terminal cytoplasmic regions (Nichols and Lopatin, 1997; Hibino et al., 2010). The subunits are organized such that one transmembrane *α*-helix (TM1) is on the outside of the channel while the other (TM2) is on the inside near the pore (Hibino et al., 2010). The pore-forming region includes a selectivity filter that confers passage of K^+^ but excludes Na^+^ (Doyle et al., 1998). In Eukaryotes, the selectivity filter is characterized by a seven-residue motif TXGYGFR, where X is an aliphatic amino acid, and F is sometimes replaced by a different residue (Tao et al., 2009).

Sixteen genes encoding Kir channel subunits have been identified in mammals. They belong to seven subfamilies (Kir1 to Kir7) comprising four functional groups: classical, G protein-gated, ATP-sensitive, and K^+^-transport channels (Hibino et al., 2010; Walsh, 2020). In mammals, Kir channels are expressed by diverse tissues comprising the nervous, muscle, cardiovascular, and urinary systems (Walsh, 2020). Consequently, abnormal function of Kir channels has been implicated in human diseases affecting these organ systems (Zangerl-Plessl et al., 2019). Mutations that cause trafficking defects associated with Kir channel dysfunction have been identified and primarily occur in two “hotspots”: in and around the TM1 domain and in a segment of the C-terminal cytoplasmic region (Zangerl-Plessl et al., 2019).

Compared to mammals, insects possess a smaller repertoire of Kir channels, ranging in number from three to six genes in the species studied thus far (Lai et al., 2020). Based on phylogenetic analysis, there are at least three different subtypes of insect Kir channels. *Drosophila melanogaster* (Luan and Li, 2012) and *Nilaparvata lugens* (Ren et al., 2018) each have three (Kir1-Kir3), while *Aedes aegypti* has four due to a duplication in Kir2 (*Kir2A* and *Kir2B*) (Piermarini et al., 2013). However, the aphids *Acyrthosiphon pisum* and *Aphis glycines* have only two (Kir1 and Kir2), suggesting a loss of Kir3 during aphid evolution (Piermarini et al., 2018). A recent study of Kir channels revealed a possible fourth subtype in Lepidoptera (Kir4), which was not found in the Diptera, Heteroptera, and Homoptera included in the study (Lai et al., 2020).

## Bioinformatics of Kir Channels in Ticks

Screening of the NCBI databases produced a single Kir channel subunit gene in 20 tick species ranging from 474 to 492 amino acids in length (**Table 1**). All are apparently full-length, having start and stop codons, except for *Ornithodoros erraticus* and *O. moubata*, which did not have stop codons. Their length, however, was similar to the Kir channels in *O. rostratus* and *O. turicata* and likely represent full-length or near full-length proteins. Kir channels in *R. annulatus* and *R. microplus* were manually annotated from their respective genome assemblies and have coding sequences with six exons spanning 7,014 and 9,318 nucleotides, respectively. Despite improvements in sequencing technologies that have led to the generation of several high-quality tick genome assemblies in recent years (Miller et al., 2018; Jia et al., 2020), the lack of genome assemblies prevented the screening of all 20 tick species for additional Kir channel genes.

**Table 1:** Summary of tick Kir channel proteins identified in the NCBI databases. All were from transcriptome shotgun assemblies (TSA database) except *Rh. annulatus* and *Rh. Microplus*, which were acquired from their respective genome assemblies.

Species	Common name	NCBI accession	Length
*Amblyomma aureolatum*		GFAC01003848	490
*Dermacentor variabilis*	American dog tick	GGTZ01000785	486
*Haemaphysalis longicornis*	Asian longhorned tick	GIKJ01016725	480
*Hyalomma excavatum*		GEFH01003741	488
*Ixodes holocyclus*	Australian paralysis tick	GIBQ01000735	474
*Ixodes persulcatus*	Taiga tick	GBXQ01023957	479
*Ixodes ricinus*	Castor bean tick	GFVZ01130232	479
*Ixodes scapularis*	Black-legged tick	GGIX01123694	479
*Ornithodoros erraticus*		GFWV01008518	492
*Ornithodoros moubata*	African hut tampan	GFJQ01004171	486
*Ornithodoros rostratus*		GCJJ01005425	485
*Ornithodoros turicata*	Relapsing fever tick	GDIE01101609	489
*Rhipicephalus annulatus*	Cattle fever tick	WOVY00000000	488
*Rhipicephalus appendiculatus*	Brown ear tick	GEDV01010278	487
*Rhipicephalus bursa*		GFZJ01000158	487
*Rhipicephalus haemaphysaloides*		GIJA01021829	488
*Rhipicephalus microplus*	Southern cattle fever tick	WOVZ00000000	490
*Rhipicephalus pulchellus*	Zebra tick	GACK01008623	488
*Rhipicephalus sanguineus*	Brown dog tick	GINV01002203	488
*Rhipicephalus zambeziensis*		GFPF01010722	487

**Figure 1:**
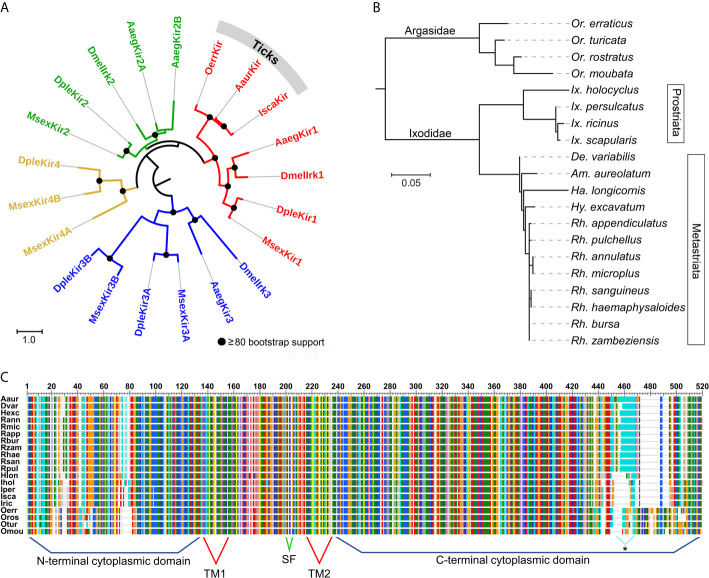
Phylogenetic relationships and protein features of tick and insect Kir channels. **(A)** Estimated phylogenetic relationships of three tick Kir channels with those in D. melanogaster (Dmel), Aedes aegypti (Aaeg), Manduca sexta (Msex) and Danaus plexippus (Dple). The multiple alignment was conducted using the L-INS-I method in MAFFT v7.475 (Katoh et al., 2019). The phylogenies **(A,B)** were estimated using the maximum likelihood method and LG substitution model in RaxML v8.2.11 (Stamatakis, 2014). Bootstrap support was estimated using 500 replications. The trees are rooted at the midpoint. **(B)** Estimated phylogenetic relationships among 20 tick Kir channel proteins. **(C)** Multiple sequence alignment of 20 tick Kir channels illustrating the predicted transmembrane (TM) and cytoplasmic domains, and the K+ selectivity filter (SF). The C-terminus of the Metastriata ticks, except Ha. longicornis (Hlon) contains long stretches of glutamine repeats. Multiple alignment conducted using MAFFT v7.475 (Katoh et al., 2019).

Based on phylogenetic analysis of the Kir channel subunits in a soft tick (*O. turicata*), two hard ticks (*I. scapularis* and *R. microplus*), two Diptera (*D. melanogaster* and *Ae. aegypti*) and two Lepidoptera (*Manduca sexta* and *Danaus plexippus*), the tick Kir channels belong to the Kir1 clade in insects (**Figure 1A**). Amino acid percent identity of tick Kirs to *D. melanogaster* Kir1 ranged from 50 to 51%. The relationships among all 20 tick Kir channel subunits are consistent with recent systematic analysis of ticks based on 18S and 28S rRNA genes and whole mitochondrial genomes (Mans et al., 2019). The soft ticks (Argasidae) and hard ticks (Ixodidae) are separated into two distinct clades, with *Ornithodoros* spp. representing the soft ticks and the remaining being hard ticks (**Figure 1B**). The soft and hard ticks differ in their feeding strategies, wherein the soft ticks are fast-feeding, and the hard ticks are long-feeding, and adaptations associated with these different feeding strategies have been described (Mans and Neitz, 2004). The divergence observed between the hard and soft tick Kir channels could reflect the physiological demands of the different feeding strategies. The Ixodidae Kir subunits formed two clades representing the Prostriata (Ixodes) and the Metastriata (all other Ixodidae), which can be separated based on morphological characteristics of the anal grooves.

A multiple sequence alignment shows the vast majority of variation to be in the N- and C-terminal domains of the protein, which flank the more highly conserved transmembrane region (**Figure 1C**). All of the tick Kir channels examined in the present study have the K^+^ selectivity filter motif of TIGYGSR, except for *O. moubata* which has the motif TIGYGFR (**Figure S1**). The C-terminal domain of the Prostriata ticks, with the exception of *Ha. longicornis*, is characterized by microsatellite repeats (CAG and CAA) coding for runs of glutamine (Q), ranging from 10 to 13 residues in length (**Figures 1C** and **S1**). The role of this highly variable region of the N-terminal domain has yet to be determined.

## Kir Channels as Targets for Novel Control Measures

Hematophagous arthropods overcome extreme physiological challenges during and after blood feeding, most notably, post-prandial diuresis (Beyenbach, 2003; Benoit and Denlinger, 2010); therefore, inhibition of renal function could provide a means to control blood feeding arthropods and the pathogens they transmit (Piermarini et al., 2017). A series of experiments aimed at understanding insect renal physiology led to the discovery of Kir channels as key components of diuresis in insect Malpighian tubules. Among these studies were gene expression analyses showing relatively high expression of Kir channels in Malpighian tubules and functional characterization of Kir1 and Kir2 subtypes using *Drosophila* S2 and *Xenopus* oocyte heterologous expression systems (Döring et al., 2002; Piermarini et al., 2013).

The identification of small molecule inhibitors has proven to be invaluable for *in vivo* and *in vitro* analyses of Kir channels and could lead to the development of novel insecticides. The small molecule inhibitors of mammalian Kir channels, VU590 and VU573, (Raphemot et al., 2011; Denton et al., 2013) were found to modulate the activity of *Ae. aegypti* Kir1 (VU590 and VU573) and Kir2B (VU573) and cause renal failure and/or mortality in *Ae. aegypti* (Piermarini et al., 2013; Raphemot et al., 2014). One of the major concerns during the development of insecticides is the effect on non-target species such as humans and beneficial insects. A small molecule inhibitor of mosquito Kir1 (VU041) was identified by high-throughput screening that does not inhibit most mammalian Kir channels (exception Kir2.1) and is not lethal to honey bees when applied topically at the concentrations tested (Swale et al., 2016). Further, VU041 was effective in reducing fecundity in insecticide resistant and wild-type strains of *Ae. aegypti* and *An. gambiae* (Swale et al., 2016).

In addition to Malpighian tubules, there is growing evidence that Kir channels perform vital functions in the salivary glands, which are essential for osmoregulation, blood feeding, and pathogen transmission in hematophagous arthropods, making them attractive targets for disruption (Ribeiro, 1987; Sauer et al., 1995; Sauer et al., 2000; Swale et al., 2017; Nuttall, 2019). Bioactive factors produced in the salivary glands enable the acquisition of a blood meal at the host interface and facilitate the transmission of tick-borne pathogens to vertebrate hosts (Kazimírová and Stibraniova, 2013).

A recent study on the role of Kir channels in tick salivary gland function provided evidence that pharmacological inhibition of these ion channels reduces the secretory activity of salivary glands in the lone star tick, *Amblyomma americanum* (Li et al., 2019). The reduced secretory capacity of the salivary gland was directly correlated with a dramatic reduction of blood ingestion during feeding. This study identified small-molecule modulators of Kir channel function (VU041, VU625, and VU688) that were previously shown to be inhibitors of mosquito Kir channels (Raphemot et al., 2014; Swale et al., 2016). Similarly, small molecule inhibitors reduced salivary gland secretion (VU041, VU590, VU937, and VU063) and blood meal ingestion (VU041 and VU063) in the horn fly, *Haematobia irritans* (Li et al., 2020). Although four inhibitors (VU041, VU590, VU937, and VU063) caused mortality, VU041 was the highest at 82 ± 11% (Li et al., 2020).

A recent study suggests an insecticide targeting a Kir channel has been developed and been in use for several years. Flonicamid (N‐cyanomethyl‐4‐trifluoromethylnicotinamide) is an insecticide that is highly effective against aphids, but not against other insects, including some coleopterans, lepidopterans, and dipterans (Morita et al., 2014). It is structurally similar to neonicotinoid insecticides, which target nicotinic acetylcholine receptors (Ren et al., 2018). However, flonicamid had no activity against nicotinic acetylcholine receptors, acetylcholine esterase or sodium channels (Morita et al., 2014). The mode of action remained elusive until recently, when a study showed flonicamid had a similar effect on the *Nilaparvata lugens* Kir1 channel as VU590 (Ren et al., 2018), which is a potent inhibitor of Kir1 in *Ae. aegypti* (Rouhier et al., 2014). Flonicamid also inhibited renal excretion in *Culex pipiens* and, taken together, suggests its mode of action is by inhibiting the insect Kir1 subtype (Ren et al., 2018). Curiously, flonicamid showed little efficacy against *Ha. longicornis* after spray application at 50 ppm concentration (Park et al., 2019). This lack of activity is likely due to a failure to inhibit the *Ha. longicornis* Kir channel, thus demonstrating the need for identification of tick Kir channel inhibitors.

## Conclusion

This review highlights the importance of Kir channels as potential targets for inhibition of renal and salivary gland function in arthropod pests and vectors of disease. Furthermore, we describe the Kir channel subunits in 20 tick species, thus providing the framework for *in vitro* functional analyses and screening of small molecule inhibitors. Recent efforts have only begun to shed light on the importance of Kir channels in tick physiology, especially their role in the regulation of blood feeding. Many gaps in our knowledge regarding the functional, temporal, spatial, and molecular characteristics of ticks still exist today. This review highlights the critical need for follow-up studies that can help elucidate these key aspects of tick Kir channel biology. The evidence for Kir channels as potential targets of insecticides and acaricides is mounting. These findings hold the potential to identifying targets for tick-borne pathogen intervention and control measures. Continued research elucidating the functional mechanisms of Kir channels and other membrane bound proteins, promises to bring an exciting new era in targeted pharmacological interventions. Although studies on the physiological role and molecular basis of Kir channel function in mammals and insects have provided critical groundwork, Kir channels in ticks remain understudied.

## Author Contributions

PS and AL conceived this work. PS, PH, and AL wrote the manuscript. PS, PH, and KB screened the NCBI databases for Kir sequences. All authors contributed to the article and approved the submitted version.

## Funding

This study was funded by the United States Department of Agriculture’s Agricultural Research Service (USDA-ARS) under project number: 3094-32000-042-00D.

## Conflict of Interest

Any mention of trade names or commercial products in this publication is solely for the purpose of providing specific information and does not imply a recommendation or endorsement by the U.S. Department of Agriculture. The USDA is an equal opportunity provider and employer.

The authors declare that the research was conducted in the absence of any commercial or financial relationships that could be construed as a potential conflict of interest.
